# Antidiabetic Effects of Flavan-3-ols and Their Microbial Metabolites

**DOI:** 10.3390/nu12061592

**Published:** 2020-05-29

**Authors:** Estefanía Márquez Campos, Linda Jakobs, Marie-Christine Simon

**Affiliations:** Department of Nutrition and Food Sciences, Nutrition and Microbiota, University of Bonn, 53115 Bonn, Germany; estefania.marquezc@gmail.com (E.M.C.); ljakobs@uni-bonn.de (L.J.)

**Keywords:** polyphenol, diabetes, flavonoids, catechins

## Abstract

Diet is one of the pillars in the prevention and management of diabetes mellitus. Particularly, eating patterns characterized by a high consumption of foods such as fruits or vegetables and beverages such as coffee and tea could influence the development and progression of type 2 diabetes. Flavonoids, whose intake has been inversely associated with numerous negative health outcomes in the last few years, are a common constituent of these food items. Therefore, they could contribute to the observed positive effects of certain dietary habits in individuals with type 2 diabetes. Of all the different flavonoid subclasses, flavan-3-ols are consumed the most in the European region. However, a large proportion of the ingested flavan-3-ols is not absorbed. Therefore, the flavan-3-ols enter the large intestine where they become available to the colonic bacteria and are metabolized by the microbiota. For this reason, in addition to the parent compounds, the colonic metabolites of flavan-3-ols could take part in the prevention and management of diabetes. The aim of this review is to present the available literature on the effect of both the parent flavan-3-ol compounds found in different food sources as well as the specific microbial metabolites of diabetes in order to better understand their potential role in the prevention and treatment of the disease.

## 1. Introduction

Diabetes can be classified into type 1 diabetes (T1D), type 2 diabetes (T2D), and gestational diabetes mellitus (GDM). Its prevalence has increased over the last decade, with 463 million people registered as suffering from it in 2019 (9.3% of the global population) [1]. In the case of T2D, whose prevalence constitutes around 90% of the total number of diabetes cases, its increase is directly related to ageing, increased urbanization, and obesogenic environments [1]. A rising prevalence of T1D has also been observed, but in this case the causes are not completely clear [2].

In general terms, glucose homeostasis involves glucose absorption in the intestine, glucose uptake and metabolism by organs and tissues, and glucose hepatic production [3]. In T2D, peripheral glucose uptake, mainly in muscle, is decreased. This, together with an increased endogenous glucose production, leads to a hyperglycemic status. Moreover, lipolysis is increased and the resulting free fatty acids (FFAs) and intermediary lipid metabolites all lead to a more pronounced glucose output, decreased glucose utilization, and impaired activity of beta cells. Pancreatic beta cells are stimulated to compensate the hyperglycemic state by secreting insulin, but this function deteriorates over time. Glucagon secretion by pancreatic alpha cells is, moreover, impaired. A deterioration in the incretin effect could be the cause of both the impaired insulin and glucagon secretion since there is an inadequate release of, or response to, the gastrointestinal incretin hormones post-prandially. Moreover, renal tubular glucose reabsorption is increased [3].

Due to the adverse effects that the most commonly used antidiabetic drugs can have [4], finding natural substances for preventing or treating T2D has become an attractive potential alternative. Flavan-3-ols, the most commonly ingested flavonoids [5], have been related to different health promoting outcomes such as the prevention of cardiovascular disease [6] and cancer [7]. Regarding their effects on T2D, epidemiological data show that some foods rich in flavan-3-ols, such as green tea, could lower the risk of the disease [8,9,10].

This review presents in vitro, in vivo, and clinical studies regarding the effects of flavan-3-ols on diabetes both in their original form and their microbial metabolites in order to better comprehend the underlying molecular mechanisms on diabetes prevention.

## 2. Search Criteria

A literature search was performed in Medline via PubMed for in vitro, in vivo, and human intervention trials published between 2005 and 2019 investigating the protective role of flavan-3-ols and their colonic metabolites on diabetes. Search terms included flavan-3-ol, flavanol, catechin, epicatechin, epigallocatechin, gallocatechin, procyanidin, theaflavin, γ-valerolactone, valeric acid, 3,4-dihydroxyphenyl propionic acid, 3-hydroxyphenyl propionic acid, 3-hydroxyphenylacetic acid, 3,4-dihydoxyphenylacetic acid, homovanillic acid, protocatechuic acid, 3-hydroxybenzoic acid, green tea, grape seed extract, cacao, diabetes, glucose, insulin, insulin resistance, beta cell, pancreas, glucagon, incretin effect, and vasodilation. In vitro and in vivo studies included both diabetic models and non-diabetic models. Only human trials with a study population presenting an impaired glucose metabolism (type 1 or type 2 diabetes mellitus, gestational diabetes, or pre-diabetes) were considered. The focus was on studies that primarily investigated effects on glucose metabolism.

## 3. Flavan-3-ols: Intake and Metabolism

Flavan-3-ols constitute a flavonoid subclass naturally present in food as monomers (catechin (C) and epicatechin (EC)), oligomers, polymers (proanthocyanidins), and other derived compounds (such as theaflavins and thearubigins) [11].

Monomeric forms of flavan-3-ols are commonly present in cocoa beans, nuts, and fruits such as berries, stone fruits, apples, and pears [12]. Cocoa, berries, and nuts are also rich in proanthocyanidins [12]. Green tea is rich in gallocatechins while fermented black and oolong teas are sources of theaflavins and thearubigins [13].

The mean flavan-3-ol intake seems to range between 77 mg/day and 182 mg/day depending on the region, representing a much higher intake than that of other polyphenols [5]. Although the intake of flavan-3-ols is the highest among other polyphenols, the amount as well as the subtype ingested differ among countries. For example, the UK was shown to be the country with the highest total flavan-3-ol consumption in Europe, which is probably due to the widespread and high consumption of tea [14]. Therefore, monomer (especially epigallocatechin-3-gallate (EGCG)) and theaflavin (TF) intake were the highest in the UK [5,14]. Nevertheless, proanthocyanidin intake was statistically higher in Mediterranean countries, with the main sources there being stone and pome fruits [5,14].

After ingestion, the monomeric forms of the flavan-3-ols are absorbed directly in the small intestine by passive diffusion before undergoing reactions lead by the phase II enzymes [11]. These enzymatic reactions, which first take place in the enterocyte and later in the liver, are performed by uridine-5’-diphosphate glucuronosyltransferases (UGT), catechol-O-methyltransferases (COMT), and sulfotransferases (SULT). The conjugated metabolites (glucuronides, O-methyl-esters, and sulphates, respectively) are then released [11]. The conjugated metabolites are water-soluble and can circulate through the human body via the systemic blood stream or be removed from the body in the urine and bile [11,15,16]. When the conjugated metabolites are eliminated via the bile, they can be recycled because they can be transported to the duodenum, where they will undergo enzymatic modifications and be reabsorbed [15].

The remaining unabsorbed ingested oligomeric and polymeric forms of flavan-3-ols, as well as a fraction of the structures already absorbed in the small intestine, go to the colon [11]. There, the microbiota can perform metabolic transformations of the flavan-3-ols aided by hydrolysis reactions (O-deglycosylation and ester hydrolysis), cleavage (C-ring cleavage, delactonization, demethylation), and reductions (dehydroxylation and double bond reduction) [17,18]. Specific colonic metabolites for flavan-3-ols are γ-valerolactones, while further phenolic compounds are also common after the microbial catabolism of other flavonoids [11].

After absorption, flavan-3-ols’ colonic metabolites go through phase II metabolism in the liver and their conjugated forms reach the organs and tissues, where they exert their potential positive effects [11]. Since the microbial metabolites could be the active substances with beneficial physiological effects in addition to their precursor compounds, flavan-3-ol-derived metabolites formed by the colonic microbiota have been given significant attention [11].

## 4. Antidiabetic Effects of Flavan-3-ols: In Vitro and In Vivo Studies

**Figure 1:**
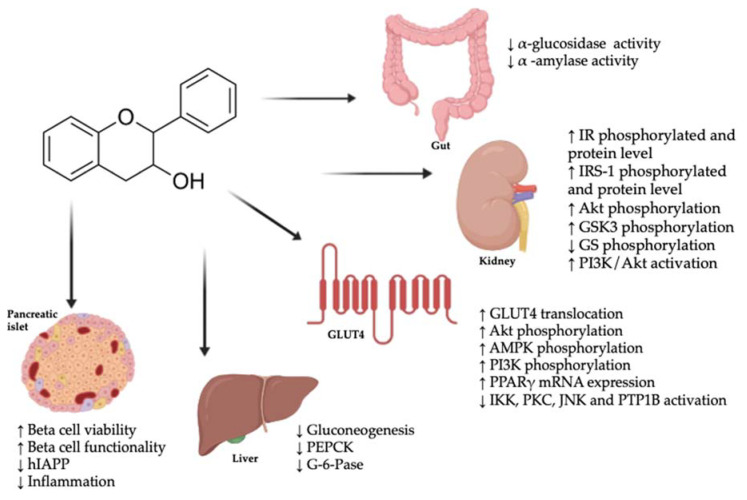
Potential molecular mechanisms underlying the antidiabetic properties of flavan-3-ols. ↑: increase; ↓: decrease; Akt: protein kinase B; AMPK: 5’ adenosine monophosphate-activated protein kinase; G-6-Pase: glucose-6-phosphatase; GLUT4: glucose transporter type 4; GS: glycogen synthase; GSK3: glycogen synthase kinase 3; hIAPP: human islet amyloid polypeptide; IKK: IκB kinase; IR: insulin receptor; IRS-1: insulin receptor substrate 1; JNK: c-Jun N-terminal kinases; mRNA: messenger RNA; PEPCK: phosphoenolpyruvate carboxykinase; PI3K: phosphoinositide 3-kinase; PKC: protein kinase C; PPARγ: peroxisome proliferator-activated receptor-γ; PTP1B: protein-tyrosine phosphatase 1B.

Flavan-3-ols and their colonic metabolites can modulate the molecular mechanisms involved in the pathogenesis of diabetes, including the glucose absorption rate in the gut, glucose peripheral uptake, glucose secretion, the modulation of beta cell function, the modulation of insulin secretion, and the modulation of the incretin effect (Figure 1).

### 4.1. Glucose Absorption in the Gut

The first factor contributing to the postprandial glycemic level in the plasma is the absorption of glucose in the gastrointestinal tract. This process is regulated by key enzymes such as α-glucosidase, which releases glucose from complex carbohydrates. Inhibition of α-glucosidase activity by a green tea water extract, a green tea polyphenol mixture, and EGCG has been shown to be stronger than by acarbose (half maximal inhibitory concentration (IC_50_) values were 4.421 ± 0.018, 10.019 ± 0.017, and 5.272 ± 0.009 µg/mL for flavan-3-ols, respectively, and 4822.783 ± 26.042 µg/mL for acarbose) [19] (Table 1). In addition, grape seed extract (GSE) (86% gallic acid equivalents) inhibited α-glucosidase activity (IC_50_ = 1.2 ± 0.2 µg/mL) more strongly than acarbose (IC_50_ = 91.0 ± 10.8 µg/mL), and of the individual catechin 3-gallates, EGCG was the one with the strongest inhibitory effect (IC_50_ = 0.3 ± 0.1 µg/mL) [20]. 

Similarly, epicatechin-3-*O*-(3-*O*-methyl) gallate (ECG3”Me), epigallocatechin-3-*O*-(3-*O*-methyl) gallate (EGCG3”Me), EGCG, and epicatechin-3-*O*-gallate (ECG) inhibited α-glucosidase, and in this case EGCG3”Me had the strongest effect. Their IC_50_ values were 14.7, 8.1, 13.3, and 61.1 µM respectively [21]. C was also shown to inhibit α-glucosidase stronger than acarbose (IC_50_ = 87.55 µg/mL vs. 199.53 ± 1.12 μg/mL, respectively) [22].

Interestingly, isolated procyanidins B2, B5 (dimeric), and C1 (trimeric) also had stronger α-glucosidase inhibitory activities than acarbose (IC_50_ = 4.7 ± 0.2, 5.5 ± 0.1, and 3.8 ± 0.2 µg/mL, versus IC_50_ = 130.0 ± 20.0 µg/mL, respectively), suggesting that the inhibitory activity could be correlated to the molecular weight of the compound [23].

For α-amylase, another digestive enzyme responsible for starch hydrolysis, GSE (86% gallic acid equivalents) inhibited its activity (IC_50_ = 8.7 ± 0.8 µg/mL), with the same potency as acarbose (IC_50_ = 6.9 ± 0.8 µg/mL) [20]. However, α-amylase was not strongly inhibited by tea extracts and individual catechin 3-gallates [20].

These effects have also been observed in mice fed with proanthocyanidins with different degrees of polymerization [52] (Table 2). Mice fed with proanthocyanidins with a high degree of polymerization showed a stronger inhibition of α-amylase activity both in the small intestine and in the pancreas than those fed with a low degree of polymerization proanthocyanidins. The rates of inhibition compared to the control group were 41% in the small intestine and 45% in the pancreas for high degree of polymerization proanthocyanidins, and 21% and 26% for low degree of polymerization proanthocyanidins [52].

### 4.2. Insulin Signaling Pathways and Glucose Peripheral Uptake

Due to the polar nature of glucose, its transport into the cell requires the use of transporter proteins in the cell membrane. These glucose transporters have different tissue distributions and a specific affinity for carbohydrates [69]. The insulin-regulatable glucose transporter type 4 (GLUT4) is found in insulin-sensitive tissues: skeletal muscle, cardiomyocytes, and adipocytes. Under physiological conditions, the insulin-mediated translocation of intracellular GLUT4 from the cytoplasm to the plasma membrane results in the uptake of glucose. This process is influenced by phosphoinositide 3-kinase (PI3K), protein kinase B (PKB or Akt), and protein kinase C zeta type (PKCζ). In short, insulin binding to insulin receptor (IR) leads to the phosphorylation of the beta subunit which, at the same time, phosphorylates the insulin receptor substrate (IRS). Upon tyrosine phosphorylation, which could be inhibited by serine phosphorylation of insulin receptor substrate 1 (IRS-1), PI3K binds to IRS and activates the Akt/PKB and the PKCζ cascades. Activated Akt induces glycogen synthesis via the inhibition of glycogen synthase kinase (GSK-3). Eventually, the Rab GTPase-activating protein AS160 (Akt substrate of 160 kDa) is activated, leading to the translocation of GLUT4 to the plasma membrane and glucose uptake [70].

When the translocation of intracellular GLUT4 to the plasma membrane is impaired, insulin resistance (IRes) takes place. T2D develops when both IRes and defects in insulin secretion occur [3].

Since approximately 80% of insulin-stimulated glucose uptake in the postprandial state takes place in the skeletal muscle, this tissue plays a key role in maintaining glucose homeostasis; therefore, many studies have focused on the effect of flavan-3-ols on GLUT4 translocation in skeletal muscle.

In vitro, a cacao liquor procyanidin (CLP) extract (1–10 µg/mL), consisting of EC, C, and other procyanidins, dose-dependently enhanced glucose uptake and promoted GLUT4 translocation to the plasma membrane of L6 myotubes after 15 min of incubation [53].

A mixture of TF, theaflavin-3-gallate (TF-3-G), theaflavin-3′-gallate (TF-3′-G), and theaflavin-3,3′-digallate (TFDG) (2.5–10 µg/mL, 24 h treatment) improved IRes induced by palmitic acid in HepG2 cells, as measured by the increase in 2-(N-(7-nitrobenz-2-oxa-1,3-diazol-4-yl)amino)-2-deoxyglucose (2-NBDG) uptake using metformin as a positive control [24]. Total GLUT4 and protein levels of GLUT4 bound to the membrane were increased by theaflavins in a dose-dependent manner [24]. They reversed the reduction of the phosphorylation level of Akt induced by palmitic acid and led to an increased phosphorylation of IRS-1 (Ser307) in HepG2 cells [24]. 

Interestingly, Ojelabi et al. showed that EGCG and ECG dose-dependently inhibited sugar uptake by glucose transporter type 1 (GLUT1), which was measured using 3-*O*-methylglucose uptake. It was found that low concentrations of the flavan-3-ols activated sugar uptake, while higher concentrations inhibited sugar uptake and noncompetitively inhibited sugar exit [25]. 

Glucose uptake in induced insulin-resistant 3T3-L1 adipocytes significantly increased after incubation with EGCG at 5 µM. Moreover, EGCG dose-dependently reversed the dexamethasone (Dex) and tumor necrosis factor (TNFα)-induced increase of c-Jun N-terminal kinases (JNK) phosphorylation levels and promoted GLUT4 translocation (1 µM) [26]. 

In vivo studies showed similar results. KK-Ay mice, when supplemented with green tea catechins (98% pure) at a low as well as at high concentrations (150 mg/kg/day and 300 mg/kg/day), showed a reduced JNK phosphorylation in adipose tissues when compared to untreated animals and an increased GLUT4 content in the plasma membrane [26].

Yamashita et al. administered a CLP extract as a single dose (250 mg/kg) to mice at the Institute of Cancer Research (ICR). After carbohydrate ingestion, CLP suppressed the hyperglycemic response and improved GLUT4 translocation in skeletal muscle [53]. In fact, the GLUT4 translocation was approximately 3.9-fold higher in comparison with the control group, who were only administered water and no glucose [53]. These results were further confirmed by a consecutive administration of a CLP-supplemented (0.5%) diet to C57BL/6 mice for 7 days, which had the same effects on skeletal muscle GLUT4 after glucose load [53]. Similarly, procyanidins (both low and high degree of polymerization, 10 mg/kg) from a CLP extract prevented hyperglycemia through the promotion of GLUT4 translocation in the skeletal muscle of ICR mice [54]. This could be explained by the significantly increased phosphorylation of 5’ adenosine monophosphate-activated protein kinase (AMPK), ß-subunit of IR (IRβ), IRS-1, and PI3K by procyanidins with both low and high degrees of polymerization [54].

After the oral administration of EC, procyanidin B2, procyanidin C1, EC-(4β–6)-EC-(4β–8)-EC-(4β–8)-EC (PA4-1), and cinnamtannin A2 (PA4–2) (10 µg/kg) to ICR mice, GLUT4 translocation in skeletal muscle significantly increased compared to in control mice [55]. Trimeric and tetrameric procyanidins significantly promoted phosphorylation of PI3K, and PA4-1 was able to significantly induce phosphorylation of Akt1 at both serine 473 and threonine 308 [55]. The latter compound was the only one able to significantly promote the phosphorylation of IRS-1 as well as increase the insulin plasma level. Similarly, all compounds significantly induced phosphorylation of AMPK [55].

In a model of T1D, streptozotocin (STZ)-induced rats were administered a green tea extract (GTE) for 12 days composed of the following catechins: C, EC, (−)-gallocatechin (GC), (−)-epigallocatechin (EGC), (−)-catechin gallate (CG), ECG, (−)-gallocatechin gallate (GCG), EGCG, and caffeine [56]. After an oral glucose tolerance test (OGTT), high blood glucose induced by STZ was significantly reduced with the GTE treatment when compared to the control group. When the possible mechanisms were investigated, the authors found that the GTE treatment increased the translocation of GLUT4 in the skeletal muscle to a normal level when compared to untreated rats. In contrast, the level of the IRß was not changed. These results imply that the green tea improved hyperglycemia in T1D rats without having an influence on insulin secretion from pancreatic beta cells, by promoting GLUT4 translocation in skeletal muscle. In addition to these findings, the degree of protein glycation induced by STZ measured by fructosamine and glycated hemoglobin (HbA1c) significantly decreased after the treatment with the GTE. This result suggests not only a protective role of green tea against the manifestation of diabetic complications but also an ability to improve those already presenting [56].

In a parallel experiment, an OGTT in KK-Ay mice was also performed, but in this case, mice were treated with GTE for 63 days (one group) or for 42 days directly after the appearance of hyperglycemia (another group). The authors found that the blood glucose after green tea intake was significantly lower when compared to the control and GLUT4 translocation in the skeletal muscle was significantly increased when compared to the control, but the level of IRß remained unaltered [56]. Another result from this experiment is the significant reduction of protein glycation and triacylglycerol by green tea [56]. 

In a study from Cremonini et al., EC supplementation (20 mg/kg) in high-fat-diet-induced obese and diabetic C57BL/6 mice improved insulin sensitivity and glucose homeostasis when compared to non-supplemented and control mice. The impairment of the insulin signaling cascade in the liver and the adipose tissue induced by the high-fat diet was prevented and the upregulation/activation of proteins which inhibit the insulin pathway (IκB kinase (IKK), protein kinase C (PKC), JNK, and protein-tyrosine phosphatase 1B (PTP1B)) was prevented [57].

Bettaieb et al. found that the supplementation of the diet of high-fructose-fed rats with EC (20 mg/kg) for 8 weeks mitigated the IRes induced by the high fructose concentrations, and it reversed both the impaired activation of the insulin signaling cascade (IR, IRS-1, Akt, and extracellular signal–regulated kinases 1/2 (ERK1/2)) as well as the upregulation of negative regulators (PKC, IKK, JNK, and PTP1B) in the liver and adipose tissue [58].

Glucose uptake has been shown to be promoted not only by the flavan-3-ols in their original form but also by some of their microbial metabolites. Specifically, 5-(3,5-dihydroxyphenyl)-γ-valerolactone promoted GLUT4 translocation in L6 skeletal muscle cells and soleus muscle by phosphorylation of the AMP-activated protein kinase (AMPK) signaling pathway both in vitro and in vivo at concentrations of 1–3 µM and 32 mg/kg, respectively [27]. At 32 mg/kg it caused suppression of hyperglycemia after an OGTT, while a higher dosage of 64 mg/kg only influenced AMPK phosphorylation [27].

Other microbial metabolites unspecific to flavan-3-ols have also been shown to modulate molecular mechanisms related to diabetes. Scazzocchio et al. investigated whether protocatechuic acid exerted an effect on glucose transport in adipocytes [28]. Incubation of the metabolite at 100 µM for 18 h with human and murine adipocytes treated with oxidized low density lipoprotein (oxLDL) significantly improved glucose uptake, GLUT4 translocation, and adiponectin secretion. These effects were observed after stimulation with insulin and also without it [28]. Glucose uptake was significantly and dose-dependently enhanced in non-oxLDL-treated human and murine adipocytes without the presence of insulin up to 40% and 60%, respectively [28]. These results indicate an insulin-like activity. A reversion of the oxLDL-induced diminishment of mRNA expression and activity of the peroxisome proliferator-activated receptor-γ (PPARγ) was also observed, and its inhibition impeded both the adiponectin and GLUT4 upregulation suggesting its implication in the insulin-like activity [28].

Both EC at 10 µM and 2,3-dihydroxybenzoic acid (2,3-DHB) at 20 µM increased IR and IRS-1 tyrosine phosphorylated and total protein levels in rat renal NRK-52E cells. In addition, phosphorylated levels of Akt and GSK-3 increased and those of glycogen synthase (GS) decreased [29]. Similarly, after treatment of renal tubular NRK-52E cells with high glucose levels and either EC at 5–20 µM or 3,4-dihydroxyphenylacetic-acid (3,4-DHPA) at 10–20 µM, the induced impairment of glucose uptake was restored. At 10 µM, EC and 3,4-DHPA increased tyrosine phosphorylated levels and total levels of IR, reversed the inhibition of the PI3K/Akt pathway involved in the insulin signaling cascade, and prevented the high-glucose-induced downregulation of AMPK phosphorylation [30].

### 4.3. Beta Cell Viability and Function

In the situation of IRes, pancreatic beta cells try to maintain glucose levels by enhancing insulin production and increasing islet size and beta cell mass. However, an increased insulin response does not mean that beta cells are functioning normally. In fact, beta cells in this situation are kept under a high workload which, when maintained over time, results in functional exhaustion, dedifferentiation, and eventually beta cell death [71]. Apoptosis of beta cells is mainly induced by glucotoxicity, lipotoxicity, and deposits of islet amyloid polypeptide (IAPP) [72,73]. 

Glucose-stimulated insulin secretion (GSIS) in the beta cell line INS-1D after treatment with catechins was studied by Kaneko et al. [31]. Both EGCG at 10 μM as well as GCG at 30 μM significantly inhibited the GSIS. Furthermore, at 100 μM they almost eliminated GSIS. EC and C did not modify GSIS at concentrations up to 100 μM. At 10 μM, EGC nearly eliminated GSIS, while GC and ECG partially inhibited it. CG did not alter GSIS at concentrations up to 100 μM. Apart from this, EGCG, and not EC, inhibited the variation of intracellular Ca^2+^ concentration. These results suggest that, at concentrations higher than physiological levels, some catechins have an inhibitory effect on GSIS, which is induced by the structure-dependent inhibition of voltage-dependent Ca^2+^-channels [31].

Supporting these results, a treatment with EC at a physiological dose of 0.3 μmol/L but not at 30 μmol/L improved GSIS of saturated fatty acid (SFA)-impaired INS-1 cells [32]. This was thought to be due to a modulation of the cell secretory capacity via the activation of the Ca^2+^/calmodulin-dependent protein kinase II (CaMKII) pathway and possibly through the GPR40 receptor [32].

In humans and animals, beta cell functionality can be measured by several methods. Some of the most commonly used methods include the homeostasis model assessment (HOMA), OGTT or intravenous glucose tolerance tests and the hyperglycemic clamp procedure [74]. The ability of flavan-3-ols to affect these has been as well assessed. In a study from Othman et al., treatment of diabetic rats with EGCG (2 mg/kg) every other day over one month significantly decreased the HOMA of insulin resistance (HOMA-IR) value and increased insulin levels when compared to untreated diabetic rats [59].

In a model where male Wistar rats were contrived to be obese through a cafeteria diet, a 21-day treatment with grape seed procyanidin extract (GSPE) at 25 mg/kg (defined composition) improved IRes measured by HOMA-IR [60]. The HOMA of beta cell function (HOMA-β) index also decreased. Insulin gene expression in the pancreas tended to decrease in treated rats, and a significant decrease in the expression of carboxypeptidase E (Cpe) was also shown [60]. On the other hand, treatment with GSPE enhanced the increase in the Bcl-2-associated X protein (Bax) levels induced by the cafeteria diet, which suggests an increased apoptosis in the pancreas in contrast to results from other studies [60]. 

Gan et al. suggested that EGCG dose-dependently improved IRes in high-fat diet non-alcoholic fatty liver disease (NAFLD) mice by enhancing the insulin clearance of the hepatic insulin degrading enzyme (IDE) [61]. In this study, NAFLD mice were administered 10, 20, and 40 mg/kg EGCG intraperitoneally. Hyperglycemia, hyperinsulinemia, and IRes observed in mice fed a high-fat diet without EGCG were reversed by the polyphenol [61].

Insulin deficiency and IRes have been described in ß-thalassemia patients with iron overload, which is probably a secondary effect of a diminished pancreatic beta cell function. The incubation of iron-loaded rat insulinoma pancreatic β-cells with a GTE (2.29 µg EGCG equivalent) increased insulin secretion levels 2.5-fold and decreased cellular levels of iron and reactive oxygen species (ROS) [33].

The effect of flavan-3-ols directly on beta cell viability was also assessed. Cinnamtannin B1, procyanidin C1, and cinnamtannin D1 from cinnamon extracts were shown to dose-dependently protect INS-1 cells from palmitic acid and H_2_O_2_-induced reduction in terms of cell viability [34]. At 25 μmol/L, they enhanced insulin secretion in lipotoxic INS-1 cells [34]. However, the flavan-3-ols EC and procyanidin B2 had no significant effects [34].

In *db*/day*b* mice, treatment with EGCG (10 g/kg diet, 1% (*w*/*w*)) for 10 weeks improved glucose tolerance and additionally increased GSIS similarly to rosiglitazone, although no significant effect was found in IRes (HOMA-IR and quantitative insulin sensitivity check index (QUICKI)). This effect may be mediated by changes in pancreatic islets, since the number and size of pancreatic islets increased, together with a reduction of islet endoplasmic reticulum stress markers ex vivo [62].

The literature suggests that human islet amyloid polypeptide (hIAPP) fibril formation contributes to T2D by causing beta cell dysfunction and apoptosis. For this reason, the inhibition of the formation of toxic hIAPP oligomers and fibrils may be a good therapeutic strategy for the management of T2D. Some authors have therefore tried to elucidate the role of flavan-3-ols in the prevention of their formation. In hemizygous non-diabetic hIAPP transgenic mice treated with EGCG (0.4 mg/mL) for three weeks, EGCG reduced amyloid fiber intensity suggesting a beneficial effect on pancreatic amyloid fibrils in vivo [63]. However, there was no effect on diabetic hIAPP transgenic mice. This, therefore, suggests that EGCG would be effective as an early therapeutic method.

Mo et al. went further and examined the molecular process by which EGCG could inhibit hIAPP aggregation [35]. The authors found that in vitro EGCG could block the inter-peptide hydrophobic/aromatic interactions responsible for inter-peptide β-sheet formation and the intra-peptide interaction related to ß-hairpin formation. Thus, the three-stranded β-sheet structures were removed and loosely packed coil-rich conformations were formed. This EGCG-induced conformational shift of the hIAPP dimer was related to hydrophobic, aromatic stacking, cation-π, and H-bonding interactions [35].

Adding to these results, Meng et al. proved that EGCG inhibited in vitro amyloid formation by IAPP and disaggregated IAPP amyloid fibrils. At the same time, EGCG protected cultured rat INS-1 cells against IAPP-induced toxicity at 30 µM [36]. EGCG (2–32 µM) was also shown to inhibit the nucleation and fibrillation of hIAPP by forming hIAPP amorphous aggregates instead of ordered fibrils [37]. Moreover, a complex of Al(III)/EGCG was able to inhibit hIAPP fibrillation more effectively than the flavan-3-ol alone [37].

T-cell-mediated destruction of pancreatic beta cells leads to insulin deficiency in T1D. In addition, inflammation is known to play a role in the pathogenesis of T1D [3]. In this regard, EGCG prevented the onset of T1D in non-obese diabetic (NOD) mice when administered at 0.05% in drinking water (60–90 mg/kg body weight (b.w.), equivalent to 4.5–6.8 g/day by a 75 kg person) for 32 weeks [64]. Compared to control mice, plasma insulin levels were higher, HbA1c concentrations were lower, and circulating anti-inflammatory cytokine interleukin 10 (IL-10) levels were increased. However, no effect on pancreatic insulitis was observed. When human pancreatic islets were incubated with inflammatory cytokines, addition of EGCG (1 and 10 µM) promoted islet viability [64]. Similarly, the administration of EC at 0.5% in drinking water (equivalent to an intake of 250 g dark chocolate containing 6% EC) for 32 weeks also delayed the development of T1D [65]. Importantly, pancreatic islet mass was preserved and the lymphatic infiltration into islets was lower meaning an improvement in the insulitis. Anti-inflammatory cytokine IL-10 levels increased [65]. HbA1c concentrations were, in this case, significantly lower and plasma insulin levels were significantly higher in mice treated with EC than in untreated mice [65]. 

The effect of low molecular weight phenolics produced after colonic metabolism of flavan-3-ols on beta cell functionality and viability has also been assessed. Fernández-Millán et al. found out a significant increase in GSIS in INS-1E pancreatic beta cells and isolated rat islets after treatment with 3,4-DHPA and 3-hydroxyphenyl propionic acid (3-HPP) at low concentrations (5 and 1 µM, respectively) [38]. Under oxidative stress induced by tert-butyl hydroperoxide (*t*-BOOH), both metabolites restored GSIS to control levels and significantly decreased cell death [38]. PKC and ERK could play a role in producing the observed effect, since their phosphorylation levels increased after treatment [38].

3,4-DHPA (250 µM) could also prevent the diminished insulin secretion induced by high cholesterol on Min6 pancreatic beta cells [39]. Moreover, it dose-dependently prevented cholesterol-induced cytotoxicity and apoptosis. Oxidative stress and mitochondrial dysfunction were also prevented [39].

5-Phenylvaleric acid, hippuric acid and homovanillic acid improved GSIS in beta cells more effectively than EC at concentrations up to 100 µM [40]. In addition to stimulating beta cell function, the microbial metabolites enhanced glucose utilization in skeletal muscle [40]. 

### 4.4. Endogenous Glucose Production

The liver’s inability to perceive insulin signals directly after glucose ingestion leads to the continuing production of glucose and, therefore, importantly contributes to a hyperglycemic status [3]. The maintained glucose output by the liver can be a consequence of two processes: gluconeogenesis and glycogenolysis [3]. However, the latter has a less important role in the increased glucose production of T2D patients [75]. The mechanisms responsible for the increase in hepatic gluconeogenesis include hyperglucagonemia, higher circulating levels of gluconeogenic precursors (lactate, alanine, and glycerol), elevated FFA oxidation, enhanced sensitivity to glucagon, and reduced sensitivity to insulin [3]. 

Increased activity of insulin-influenced phosphoenolpyruvate carboxykinase 1 (PCK1) and glucose-6-phosphatase (G-6-Pase) seems to contribute to the accelerated rate of hepatic glucose production [3]. In this sense, studies have shown how flavan-3-ols affect the expression of key regulators of the gluconeogenesis pathway. 

Waltner-Law et al. studied the effects of green tea compounds on insulin signaling pathways, gene expression, and glucose production [41]. The authors found that EGCG had insulin-like activities in hepatoma cells. At 25 µM, EGCG reduced glucose production to basal levels in a similar way to insulin (10 nM) and these effects were already significant at lower concentrations (12.5 µM). When studying the impact of the flavan-3-ol on the expression of genes encoding gluconeogenic enzymes, EGCG reduced phosphoenolpyruvate carboxykinase (PEPCK) mRNA in a dose-dependent manner (12.5–100 µM) and both PEPCK mRNA and G-6-Pase in a phosphoinositide 3-kinase (PI3K)-dependent manner [41]. In addition, 50 µM EGCG could activate PI3K within 10 min, similar to insulin (10 nM), but the activation of other kinases such as PKB and p70s6k was much slower and not significant. The authors suggested that EGCG has a similar mechanism to insulin in reducing glucose production and expressing the PEPCK and G-6-Pase genes by modulation of the redox state of the cell [41].

Smaller amounts of EGCG (0.25–1 µM) suppressed gluconeogenesis in mouse cyclic adenosine monophosphate dexamethasone (cAMP-Dex)-stimulated hepatocytes and blocked the expression of the PEPCK and G-6-Pase genes [42]. However, no effect on the stimulation of tyrosine phosphorylation of IRS-1 or Akt, nor an influence of the PI3K inhibitor LY294002, was found suggesting an independent mechanism to the insulin signaling pathway [42]. The other known suppressor of hepatic gluconeogenesis, apart from the insulin signaling, is AMPK. In this case, EGCG increased the AMPK and acetyl-CoA carboxylase (ACC) phosphorylation in a time- and dose-dependent manner, and the suppression of AMPK resulted in the reversion of the effect of EGCG on the expression of the PEPCK and G-6-Pase genes in a calcium/calmodulin-dependent protein kinase kinase (CaMKK)- and ROS-dependent manner [42].

Yadollah et al. showed that 40 μM EGCG significantly reduced the expression of PEPCK and G-6-Pase in insulin-resistant HepG2 cells by 53% and 67%, respectively [43]. This effect was similar to that of 10 μM pioglitazone, which is a medication used to treat T2D. A combination of EGCG and pioglitazone induced a stronger reduction in the expression of PEPCK and G-6-Pase. The authors also proved that glucose production in HepG2 cells was significantly reduced by 50% by EGCG, by 55% by pioglitazone, and by 69% by a combination of both EGCG and pioglitazone [43].

Aside from the liver, the kidneys are also involved in glucose homeostasis and gluconeogenesis. EC (5–20 µM) and 2,3-DHB (20 µM) reduced cellular glucose uptake in rat renal NRK-52E cells similarly to the sodium-glucose cotransporter-2 (SGLT-2) antagonist phlorizin, leaving the expression of SGLT-2 and glucose transporter type 2 (GLUT2) unaltered [29]. A reduction in glucose production and PEPCK levels was also observed [29]. Moreover, the authors showed that Akt was involved in the modulation of both PEPCK levels and glucose production in NRK-52E cells [29].

Treatment of renal tubular NRK-52E cells with EC (10–20 µM) and 3,4-DHPA (10 µM) separately alleviated the alterations in glucose production and the upregulation of PEPCK induced by high glucose [30]. However, the protective effect disappeared when Akt and AMPK were inhibited. Therefore, both Akt and AMPK seem to be key molecules in the modulation of the glucose homeostasis and the preservation of renal tubular functionality [30].

### 4.5. Incretin Effect

Incretin hormones include glucose-dependent insulinotropic polypeptide (GIP) and glucagon-like peptide-1 (GLP-1). They are gut peptides secreted after the intake of nutrients, such as glucose, and are responsible for the incretin effect, which is the increased stimulation of insulin secretion by oral glucose rather than by intravenous glucose infusion. This effect is impaired in patients with T2D due to the reduced insulinotropic effect of GIP and GLP-1 [76]. In addition to the insulinotropic activity, the incretin hormones work together to regulate glucagon secretion: GIP stimulates glucagon secretion while GLP-1 inhibits glucagon secretion by alpha cells. In diabetic patients, glucagon secretion is altered since it is not inhibited in hyperglycemic conditions [70].

Yamashita et al. studied if isolated dimeric, trimeric, and tetrameric procyanidins from cacao liquor administered as a single-dose (10 µg/kg) in mice could influence GLP-1 and insulin levels in plasma [66]. The tetrameric procyanidin cinnamtannin A2 was the only compound able to increase the plasma insulin level without a glucose load as well as significantly increase the GLP-1 secretion levels in plasma 60 min after oral administration [66]. In vitro experiments revealed an increased phosphorylation of proteins IRß and IRS-1 in the soleus muscle as a result of the action of insulin. Procyanidins (low and high degree of polymerization, 10 mg/kg) from a CLP-rich extract increased GLP-1 secretion with or without glucose load in mice [54].

González-Abuín et al. evaluated the modulation of the mechanisms that have an influence on GLP-1 secretion in STC-1 cells by GSPE [44]. The authors found out that 0.05 mg/L GSPE induced depolarization, while 50 mg/L induced hyperpolarization in enteroendocrine cells [44]. This high extract concentration suppressed GLP-1 secretion by around 40%. Under nutrient-stimulated conditions, 50 mg/L GSPE reduced the membrane depolarization induced by nutrients and reduced GLP-1 secretion by 20% in glucose- and proline-stimulated cells. These results indicate the importance of the GSPE concentration in depolarization and GLP-1 secretion by STC-1 cells, as well as the influence that nutrients have on GLP-1 secretion by enteroendocrine cells [44].

Glycogen synthesis is also one of the functions of incretin hormones. Its secretion rate in muscle is controlled by GS, which is also enhanced by insulin. Therefore, this stimulates a cascade of phosphorylation-dephosphorylation reactions [3]. Glycogen synthase phosphatase (PP1) is activated by the phosphorylation of serine phosphorylation site 1 in the regulatory subunit (G) of PP1 by insulin and this phosphorylation is catalyzed by insulin-stimulated protein kinase 1 (ISPK-1). Phosphorylation of site 2 by cAMP-dependent kinase (PKA) leads, on the contrary, to its inactivation [3].

Some authors have studied how flavan-3-ols influence glycogen synthesis. Kim et al. showed that green tea polyphenols consisting of 68% EGCG were able to enhance glycogen synthesis by up to a factor of 2 (10 µM) in high glucose treated HepG2 cells under 100 nM insulin stimulation [45]. The molecular mechanism can involve the regulation of enzymes such as glycogen synthase kinase 3-beta (GSK3ß) and GS since expression of phospho-GSK3β (Ser9) and phospho-GS (Ser461) were enhanced by EGCG [45].

### 4.6. Other Mechanisms

The production of cellular oxidants may affect insulin sensitivity via the negative regulation of insulin signaling pathways (JNK, IKK), the promotion of sustained chronic inflammation, and oxidative stress. Flavan-3-ols are known to have antioxidative functions, and these could exert a protective effect against diabetes and its complications via controlling the oxidative stress. Cinnamtannin B1, procyanidin C1, and cinnamtannin D1 (12.5–50 µmol/L) from cinnamon extracts inhibited H_2_O_2_-induced ROS generation as well as increased cell viability of INS-1 cells [34]. Similarly, under *t*-BOOH-induced oxidative stress, the microbial metabolites 3,4-DHPA and 3-HPP (5 and 1 µM, respectively) significantly decreased rat pancreatic beta cell death and ROS and carbonyl group production [38]. While EC at a low dose of 0.3 μmol/L, but not at a higher dose of 30 μmol/L, improved GSIS of SFA-impaired INS-1 cells, only the highest dose of EC significantly reduced ROS after treatment with H_2_O_2_ and high glucose [32].

Bettaieb et al. found that the supplementation of high-fructose-fed rats with EC (20 mg/kg) for 8 weeks, mitigated the IRes induced by high fructose concentrations. EC supplementation (20 mg/kg) in high-fructose-fed rats showed an ability to inhibit the expression and activity of NADPH oxidase and the activation of redox-sensitive signals [58].

Treatment of STZ-induced diabetic rats with C (20 and 40 mg/kg/day) significantly decreased glucose levels, while superoxide dismutase (SOD), catalase (CAT), and glutathione S-transferase (GST) levels increased in a concentration-dependent manner, especially after treatment with 80 mg/kg/day [67].

Haidari et al. showed that a GTE given to STZ-induced diabetic rats at 200 mg/kg for 4 weeks, significantly decreased their serum glucose levels as well as the serum and hepatic malondialdehyde (MDA) concentration when compared to the diabetic control group. Total antioxidant capacity (TAC) was significantly increased after treatment [68].

Plasma glucose levels could also be controlled by the modulation of lipid digestion and the reduction of hyperlipidemia [77]. C treatment of STZ-induced diabetic rats dose-dependently decreased the serum levels of total cholesterol (TC), triglycerides, LDL, apoprotein B, and glucose levels, while it increased the serum levels of high density lipoprotein (HDL) and apoprotein A-I (20–80 mg/kg) [67]. EGCG dose-dependently reversed increased serum lipid levels including TC, TG, and LDL, and increased HDL in high-fat diet NAFLD mice compared with control mice [61]. EC (20 mg/kg body weight) prevented the high-fat-diet-induced increase in plasma TG and FFA in C57BL/6 mice [57]. Treatment of HepG2 cells with 100 nM insulin and 0.1–10 µM EGCG reduced lipogenesis to 65% compared to cells treated with insulin alone through increased expressions of phosphor-AMPKα and phosphor-ACC [45].

Inflammation contributes to impaired glucose management by adipocytes, hepatocytes, and muscle cells and interferes with insulin production and insulin signaling [78]. TNFα plays an important role in the activation of signaling cascades in adipocytes related to inflammation and IRes. In this context, EC (0.5–10 µM) has been shown to dose-dependently reduce TNFα-mediated JNK, ERK1/2, and *p*-38 phosphorylation, and nuclear AP-1-DNA binding in 3T3-L1 adipocytes [46]. It also inhibited the activation of the nuclear factor kappa-light-chain-enhancer of activated B cells (NF-κB) signaling cascade preventing the p65 nuclear transport and nuclear NF-κB-DNA binding. Moreover, EC reversed the TNFα-mediated downregulation of PPARγ expression and reduced nuclear DNA binding. The altered transcription of genes involved in inflammation and insulin signaling (monocyte chemoattractant protein 1 (MCP-1), IL-6, TNFα, resistin and protein-tyrosine phosphatase 1B) mediated by TNFα was inhibited by EC [46].

EC supplementation (20 mg/kg) in high-fructose-fed rats inhibited the expression of NF-κB regulated pro-inflammatory cytokines and chemokines [58].

The colonic metabolites of flavan-3-ols have also been shown to exert beneficial effects in diabetes other than those directly related to glycemia. One of them is the positive effect on vascular function, which is known to be directly linked to diabetes [79]. As reported in several studies, low molecular weight phenolics such as 2,3-dihydroxybenzoic acid (2,3-DHB), 3-HPP, and 3,4-DHPA could exert vasodilatory activities by stimulating NO production [47,48,49]. Apart from this, dihydroferulic acid, 3-hydroxyphenylacetic acid (3-HPA), 3,4-DHPA, and homovanillic acid could reduce the formation of advanced glycation end-products (AGEs) [50,51], which are thought to be linked to the development of diabetes and insulin resistance and to the occurrence of diabetic complications [80].

## 5. Antidiabetic Effects of Flavan-3-ols: Clinical Intervention Trials

Some authors have studied the effect of the supplementation of pure flavan-3-ols on antidiabetic effects in order to exclude potential interactions with other compounds and with other flavonoids present in flavan-3-ol-rich food. Zhang et al. investigated the effects of a daily intake of EGCG (500 mg/day) in women with a diagnosed GDM at the beginning of the third quarter of pregnancy (29 weeks). HOMA-IR, HOMA-β fasting blood glucose and insulin levels decreased whereas the insulin sensitivity as measured by QUICKI increased due to the intervention. Furthermore, neonatal complications at birth, such as low birth weight or hypoglycemia, were significantly reduced in the intervention group [81] (Table 3). 

Hsu et al. found no statistical differences in several parameters (fasting glucose, insulin, HOMA-IR, HbA1c, lipoproteins, hormones (leptin, ghrelin, adiponectin), blood pressure, anthropometrics) between a decaffeinated GTE-supplemented group (3 × 500 mg/day; 856 mg EGCG) of T2D obese patients and the placebo group. However, 16 weeks of treatment led to a significant reduction of HbA1c, HOMA-IR index and the insulin level from the baseline to the end of the treatment (within-group changes) [84].

A randomized controlled trial (RCT) assessed the effect of a daily consumption of 100 g of flavanol-rich dark chocolate (FRC; 1008 mg total phenols and 36.12 g C) for 15 days on IRes and showed a significant reduction of HOMA-IR, an enhancement of the insulin sensitivity and an increase in the beta cell activity in hypertensive individuals with impaired glucose tolerance [82]. Furthermore, the consumption of FRC decreased TC and LDL when compared to the baseline values and the control, but it did not affect HDL and TG. High-sensitivity C-reactive protein (hsCRP) did not change either [82]. Similarly, the daily consumption of 27 g FRC (850 mg flavan-3-ols and 90 mg EC) and 100 mg isoflavones for one year reduced HOMA-IR, LDL, and the TC:HDL ratio and increased QUICKI and the HDL:LDL ratio in postmenopausal women with T2D. These metabolic improvements resulted in a lower 10-year total coronary heart disease (CHD) risk compared to the control [83].

A clinical trial investigating the effects of the daily intake of green tea (340 mL) and GTE (582.8 mg catechins/day) for 12 weeks showed an increased insulin level and an increase in the adiponectin level (only within-group changes) in subjects with T2D. Furthermore, there was a reduction of FFA compared to the baseline and a decrease of the TC level when compared to the control. Fasting blood glucose and HbA1c remained unchanged [85]. 

The daily supplementation with a GTE (one packet/day; 544 mg polyphenols, 456 mg C) for two months improved the HbA1c value in individuals with glucose abnormalities when compared to the baseline. No other parameters of glucose metabolism (fasting blood glucose, insulin, HOMA-IR) or lipid metabolism (TC, LDL, HDL, TG) were affected by the intervention [86]. Furthermore, the daily intake of a GTE powder did not improve the hsCRP level [86]. In individuals with borderline T2D or T2D, GTE (544 mg polyphenols, 456 mg C) decreased the IRes, as measured by HOMA-IR, the fasting blood glucose, the insulin levels and the HbA1c when compared to the baseline. No significant differences between the intervention and the control were observed [87].

However, not all studies showed unambiguous protective effects against diabetes. A daily intake of 9 g green tea in 900 mL hot water for four weeks did not affect the IRes, the fasting blood glucose or the insulin concentration in subjects suffering from T2D. Furthermore, no beneficial effects on the lipid metabolism, hsCRP or IL-6 were shown [88]. Similarly, daily supplementation of GSE (2 × 300 mg/day) for four weeks did not improve the IRes [89]. Fasting blood glucose, insulin level, and HOMA-IR remained unchanged in individuals with T2D and a high cardiovascular risk. Moreover, the supplementation did not result in an improved lipoprotein status apart from a decrease in TC level. However, the regular intake of the GSE significantly decreased fructosamine concentration, decreased hsCRP, and increased the reduced glutathione (GSH) compared to the baseline value. Total antioxidant status (TAOS) and the concentration of oxidized glutathione (GSSG) remained unchanged [89].

A daily intake of 2.5 g cacao powder (ACTICOA TM; 207.5 mg flavanols) for 12 weeks did not enhance the glucose or lipid metabolism in T2D hypertensive patients [90]. The consumption of two cacao beverages per day (2 × 28 g cacao powder/day) containing 180 mg, 400 mg or 900 mg flavanols on five consecutive days did not affect fasting and postprandial glucose parameters in obese individuals who were at risk of IRes either. However, hsCRP, 8-isoprostane, and IL-6 decreased as the dose of flavanols increased. These effects were only significant when compared to the baseline values but not when compared to the control [91].

Acute cacao studies also showed no distinct improvement of postprandial glycemia and the insulin response in participants with T2D. The acute supplementation of a cacao beverage (960 mg polyphenols; 480 mg flavanols) with a high-fat fast-food-style breakfast (766 kcal, 50 g fat) elicited a higher insulin response and a decreased IRes, as measured by HOMA-IR. HDL increased while the concentration of TC, LDL, TG, and hsCRP remained unchanged [92]. No effects could be observed after an acute supplementation of 2.5 g cacao powder (ACTICOA^TM^; 40.4 mg EC) with a diabetic-suitable breakfast in hypertensive, overweight or obese subjects with T2D [93].

## 6. Concluding Remarks

Increasing evidence suggests that flavan-3-ols are responsible for the protective role of certain foods, such as green tea, against diabetes. Possible molecular mechanisms by which they could prevent or treat diabetes include the promotion of beta cell functionality and viability, the amelioration of glucose transport in muscle and adipose tissue by the promotion of the insulin signaling pathway, the enhancement of the incretin effect, and the decrease of endogenous glucose production.

Microbial metabolites of flavan-3-ols are suggested to be the actual active form by which these compounds exert their potential health benefits, such as the antidiabetic effect. However, the evidence regarding this is still scarce and only few studies assessed the effects of flavan-3-ol specific microbial metabolites.

One of the determinants of the possible antidiabetic effect of flavan-3-ols seems to be their concentration. In this regard, when interpreting the results of in vitro studies, it is necessary to consider not only the bioavailability of the compounds investigated but also their physiological plasma concentration after absorption. Some of the studies used higher concentrations than those found in human plasma after ingestion. Conjugated flavan-3-ols have been detected in plasma in low nanomolar ranges [94,95]. Their colonic metabolites phenyl-γ-valerolactones and phenylvaleric acids have been detected in plasma at concentrations under 1 µM [95,96,97,98,99]. The lower weight phenolics are usually found in plasma at concentrations lower than 0.5 µM [96,97,98,99], although phenylacetic acid, protocatechuic acid, and hippuric acid were also detected at concentrations ≈ 40 µmol/L [96,97,98,99,100].

Although many studies used physiological concentrations of the compound and microbial metabolites, some tested higher concentrations and suggested that, in some cases, supraphysiological ranges could induce an opposite response to that from lower ranges. A careful evaluation of the flavan-3-ol dose would, therefore, be needed when used as nutraceutical.

Under physiological concentrations, flavan-3-ols and their microbial metabolites exert different biological activities at varying concentrations. The concentration needed to exert a specific function by a particular compound might not be the same than the one needed to exert another biological activity. For example, 3,4-DHPA IC_50_ on vasodilation is lower than on AGEs formation [101,102]. In the prevention and management of diabetes, low concentrations of flavan-3-ols and their microbial metabolites could influence determined molecular mechanisms while higher concentrations could be needed to positively influence other mechanisms, as shown in many of the presented studies.

Although increasing evidence supports the stated mechanisms of action, the results of many studies are inconclusive, with some of them exhibiting contradictory outcomes or even negative effects. Possible reasons for the varying results could be not only the different compound concentrations used, but also the different methodologies used in each study. As for the in vitro and animal studies, a variety of models for diabetes was used. In the literature described in this review, the most frequent in vitro techniques used insulin-secreting cell line INS-1 and pancreatic beta cell lines, and they also included in vitro studies on glucose uptake mainly in skeletal muscle cells but also in 3T3-L1 adipocytes. In addition, assays on α-amylase inhibition and inhibition of α-glucosidase activity were performed. In vivo studies included spontaneous diabetic obese animal models, mice genetically predisposed to obesity and T2D, and others used chemicals to induce the disease, mainly streptozotocin. However, not all models were specific for diabetes mellitus. Therefore, although many of these studies showed positive effects on diabetic parameters, concluding that they are beneficial for the treatment of diabetes mellitus would not be appropriate. For the proper elucidation of the effect of a substance regarding diabetes management suitable models are required. For this reason, more in vitro and animal studies using the adequate models for the disease should be performed.

Moreover, it is worth mentioning that some of the used treatment samples often include other bioactive components. Therefore, the flavan-3-ol fraction does not always represent the exclusive component present in the sample, which must be taken into account when attributing positive effects to these compounds. 

In the case of human clinical trials, the compliance of the patients to the treatment was not measured in all cases and the diet during the intervention was not always recorded. In addition, other confounders, such as body composition, were often insufficiently registered. Therefore, these factors could have influenced the studies’ results. Moreover, not only the concentration of the flavan-3-ols might be a determinant of the possible antidiabetic effect, but also, the duration of the intervention could be a determinant. Further methodological weaknesses in some of the human trials presented are the absence of a wash-out phase, or not choosing the dietary restrictions of the control groups appropriately.

It is known that the individual gut microbiota composition has an influence on both the bioavailability and the metabolization of flavan-3-ols [3]. However, none of the included clinical trials investigated the bioavailability and the metabolization of the ingested flavan-3-ols in the study population. Therefore, no conclusion can be reached about whether there is a positive association between the blood concentration of the flavan-3-ols or their metabolites, the administered dose, and the putative effect. That is, it is not possible to know to what extent different metabolic effects are related to a different bioavailability and metabolization of the flavan-3-ols by individuals. In order to better understand the effects of flavan-3-ols and their metabolites on the prevention and management of diabetes, it is relevant to record not only metabolic parameters after treatment but also the pharmacokinetics of these substances.

For all these reasons, the use of homogeneous and more appropriate methods is essential for the clarification of flavan-3-ol’s antidiabetic effect and mechanisms of action.

## Figures and Tables

**Table 1:** In vitro studies on antidiabetic effect of flavan-3-ols and their microbial metabolites ^1^.

In Vitro Test	Flavan-3-ol	Concentration/Dose	Results	Ref.
**Glucose absorption in the gut**				
Inhibition of α-glucosidase and α-amylase activity	GTE, GTP, EGCG	α-amylase: IC_50_ = 1370.812 ± 59.081–1849.612 ± 73.475 µg/mL α-glucosidase: IC_50_ = 4.421 ± 0.018–10.019 ± 0.017 µg/mL	Inhibition of α-glucosidase by GTE was stronger than by acarbose (IC_50_ = 4822.783 ± 26.042 µg/mL) and the other compounds but had no effect on α-amylase.	[19]
			Combination of GTE, GTP, EGCG, and acarbose at low concentrations had synergistic suppressive effects on α-glucosidase.	
			α-amylase was inhibited at high concentrations of GTP and EGCG, but lower than that of acarbose (IC_50_ = 2715.654 ± 24.709 µg/mL).	
Inhibition of α-amylase and α-glucosidase activity	GSE, tea extracts, C, EC, EGC, EGCG, GCG, ECG	α-amylase: IC_50_ = 8.7 ± 0.8–378 ± 134 µg/L α-glucosidase: IC_50_ = 0.3 ± 0.1–31 µg/L	α-amylase was only inhibited by GTE extract similarly to acarbose.	[20]
			α-glucosidase was significantly inhibited by all compounds except C, EC following this order: Teavigo^®^ > EGCG > GTE> GSE > GCG > WTE > ECG	
Inhibition of α-glucosidase activity	EGCG, ECG, EGCG3”Me, ECG3”Me	IC_50_ = 8.1−61.1 µM	Inhibition of α-glucosidase EGCG3”Me > EGCG > ECG3”Me > ECG	[21]
α-glucosidase inhibition assay	C	IC_50_ = 87.55 µg/mL	The α-glucosidase inhibitory potency was greater than acarbose (IC_50_ = 199.53 µg/mL).	[22]
Inhibition of α-glucosidase activity	Procyanidins B2, B5 and C1	IC_50_ = 4.7 ± 0.2, 5.5 ± 0.1 and 3.8 ± 0.2 µg/mL	Trimeric procyanidin (C1) exerted the strongest inhibitory activity. Inhibitory effect was stronger than for acarbose (130.0 ± 20.0 µg/mL).	[23]
**Insulin signaling pathways and glucose peripheral uptake**				
Glucose uptake assay and insulin signaling pathway in HepG2 cells treated with PA	Theaflavin mixture (TF, TF-3-G, TF-3′-G, and TFDG)	2.5–10 μg/mL	Increased 2-NBDG uptake. Increased membrane bound GLUT4 protein level and Akt phosphorylation. Decreased IRS-1 phosphorylation at Ser307. Increase of mtDNA copy number. Downregulation of PGC-1β mRNA level and increase of PRC mRNA expression.	[24]
GLUT1-mediated uptake of 3-*O*-methylglucose in human red blood cells	EGCG and ECG	-	Uptake of 0.1 mM 3MG was dose-dependently inhibited.	[25]
Glucose uptake, GLUT4 translocation, and JNK phosphorylation in insulin resistant 3T3-L1 adipocytes	EGCG	0.1–5 µM	At 5 µM, increased glucose uptake. Dose-dependent reversion of Dex- and TNFα-induced JNK phosphorylation. At 1 µM, increased GLUT4 translocation.	[26]
GLUT4 translocation in L6 skeletal muscle cells	5-(3,5-dihydroxyphenyl)-γ-VL	1 and 3 µM	3 µM promoted the strongest effect on GLUT4 translocation. AMPK phosphorylation increased.	[27]
Glucose transport in human and murine 3T3-L1 adipocytes stimulated or not with insulin	PCA	100 µmol/L	Reversion of oxLDL-induced decrease in glucose uptake and GLUT4 translocation. Reversion of oxLDL-induced decrease of adiponectin mRNA expression and secretion, and of PPARγ mRNA expression and activity.	[28]
Insulin signaling, glucose uptake, and glucose production in rat renal NRK-52E cells	EC, 2,3-DHB, 3,4-DHPA, 3-HPP and VA	5–20 µM	Glucose uptake, glucose production, and PEPCK reduced after treatment with EC (5–20 µM) and 2,3-DBH (20 µM).	[29]
			IR and IRS-1 phosphorylated and total protein levels increased at 10 µM EC and 20 µM 2,3-DHB. Increased phosphorylation of Akt and GSK3. The inhibition of the PI3K/Akt pathway was restrained.	
Insulin signaling and glucose uptake and production in rat renal NRK-52E cells treated with high glucose	EC, 3,4-DHPA, 2,3-DHB and 3-HPP	5–20 µM	The altered glucose uptake and production caused by high glucose was prevented by EC (5–20 µM) and 3,4-DHPA (10–20 µM). At 10 µM, tyrosine phosphorylated, and total levels of IR increased. The PI3K/Akt pathway and AMPK were activated and the PEPCK expression was reduced.	[30]
**Beta cell viability and function**				
GSIS in INS-1 cell. [Ca2+] oscillations induced by glucose in INS-1 cells	EGCG, GCG, EC, C, EGC, GC, ECG, CG	10–100 μM	GSIS was decreased by 10 and 30 μM EGCG. GSIS was terminated by 100 μM EGCG and 100 μM GCG. EGC nearly abolished GSIS at 100 μM, GC and ECG partly inhibited it. EC, C, and CG did not show any effect. 100 μM EGCG decreased the oscillation of intracellular calcium.	[31]
GSIS in SFA-treated INS-1 cell; ROS production in high-glucose and H_2_O_2_-treated INS-1 cell	EC	0.3 μmol/L 30 µmol/L	Increase of GSIS. Reversion of SFA-induced inhibition of CaMKII phosphorylation. Reduced ROS production.	[32]
Insulin production in iron-loaded RINm5F pancreatic cells. Iron and ROS levels in RINm5F pancreatic cells	GTE	1–20 µM EGCG 1–10 µM EGCG	Dose-dependent increase of insulin secretion.	[33]
			Dose-dependent decrease of iron and ROS levels.	
Cell viability and GSIS in PA- and H_2_O_2_-treated INS-1 pancreatic beta cells. H_2_O_2_-stimulated ROS production	Cinnam-tannin B1, procyanidin C1, cinnam-tannin D1	12.5–100 μmol/L	Dose-dependent increase of cell viability.	[34]
			GSIS increase at 25 µmol/L.	
			Decreased ROS production.	
Inhibition of hIAPP aggregation and molecular mechanism	EGCG	-	Blockage of inter-peptide hydrophobic/aromatic interactions and intra-peptide interactions.	[35]
			Abolishment of β-hairpin-containing three-stranded β-sheet conformation.	
			Shift of hIAPP dimer toward loosely packed coil-rich conformations.	
Amyloid formation by IAPP and disaggregation of amyloid fibrils with thioflavin-T binding assay and TEM. Cell viability in mixture IAPP:EGCG on rat INS-1	EGCG	3.2–32 µM	At 32 µM, inhibition of amyloid formation by IAPP. IAPP:EGCG (3.2 µM) complex did not seed amyloid formation by IAPP. Disaggregation of IAPP. Increased cell viability of INS-1 cells to 77%.	[36]
hIAPP fibrillation and aggregation	EGCG	2–32 µM	Inhibition of hIAPP fibrillation.	[37]
			Formation of amorphous aggregates instead of ordered fibrils.	
Beta cell function of rat INS-1E pancreatic beta cells and rat pancreatic islets	3,4-DHPA, 2,3-DHB and 3-HPP	1–5 µM	3,4-DHPA and 3-HPP enhanced GSIS (5 and 1 µM, respectively). Under oxidative stress, 3,4-DHPA and 3-HPP reduced ROS and carbonyl group production, and GSIS returned to control levels. PKC and ERKs phosphorylation improved.	[38]
Beta cell function of Min6 pancreatic beta cells incubated with cholesterol	3,4-DHPA	10–250 µM	3,4-DHPA reversed the diminished insulin secretion induced by cholesterol. It protected beta cells against apoptosis, oxidative stress, and mitochondrial dysfunction.	[39]
Beta cell function and glucose utilization in rat INS-1 beta cells and human skeletal muscle	EC, HA, HVA and 5-PVA	5–100 µM	EC (10 and 25 µM), HA, and 5-PVA (25 µM) provoked glucose oxidation in skeletal muscle. After oxidative insult, skeletal mitochondrial function was conserved. In beta cells, EC (100 µM) and metabolites (5–100 µM) stimulated GSIS.	[40]
**Endogenous glucose production**				
Glucose production and PEPCK/G-6-Pase gene expression in H4IIE rat hepatoma cells incubated with pyruvate and lactate	EGCG	12.5–100 µM	At 25 µM, glucose production was repressed comparable to that of insulin.	[41]
			Dose-dependent reduction of PEPCK mRNA as well as G-6-Pase. PI3K inhibitor LY 294,002 reversed the repression of EGCG on PEPCK and G-6-Pase gene expression. NAC and SOD reversed the increased protein-tyrosine phosphorylation and reversed PEPCK and G-6-Pase gene repression.	
Gluconeogenesis and PEPCK/G-6-Pase gene expression in mouse cAMP-Dex-stimulated hepatocytes	EGCG	0.25–1 µM	Dose-dependent attenuation of gluconeogenesis. Expression of PEPCK and G-6-Pase genes was blocked.	[42]
			Activation of AMPK mediated by CaMKK and ROS-dependent.	
Gluconeogenesis pathway in palmitate-induced insulin resistant HepG2 cells	EGCG	40 μM	Expression of PEPCK and G-6-Pase was reduced by 53% and 67%, respectively. Glucose production was reduced by 50%.	[43]
**Incretin effect**				
Plasma membrane potential and GLP-1 secretion in STC-1 cells under basal and nutrient-stimulated conditions	GSPE	0.05–50 mg/L	At 0.05 and 0.5 mg/L, membrane depolarization. At 50 mg/L, hyperpolarization and suppression of GLP-1 secretion.	[44]
			Under nutrient-stimulation, 50 mg/L limited membrane depolarization and reduced GLP-1 secretion.	
Insulin-stimulated glycogen synthesis and lipogenesis in high-glucose treated human hepatoma HepG2 cells	GTP (60% EGCG)	0.1–10 µM	Enhanced glycogen synthesis, increased phosphorylation of Ser9 GSK3ß and Ser641 GS.	[45]
			Inhibition of lipogenesis through enhanced expression of phosphorylated AMPKα and acetyl CoA carboxylase.	
**Inflammation**				
TNFα-induced activation of NF-κB, MAPKs, AP-1, and PPARγ in differentiated white 3T3-L1 adipocytes	EC	0.5–10 µM	Dose-dependent decrease of JNK, ERK1/2, and *p*-38 phosphorylation, and nuclear AP-1-DNA binding. Inhibition of NF-κB signaling cascade activation, preventing p65 nuclear transport and nuclear NF-κB-DNA binding. Altered transcription of genes (MCP-1, IL-6, TNFα, resistin, PTP1B). Attenuation of decreased PPARγ expression.	[46]
**Vasodilation**				
Vasodilation of pre-contracted isolated rat aortic rings	3-PP, 4-HPP, 3,4-DHPP, 4-HPA, 3,4-DHPA, HVA, 3-HB, PhG, 4-MC, *m*-CoA, 3-HPP and 3-HPA	100 nM	3-HPP had the strongest vasodilatory activity, which was NO and endothelium-dependent.	[47]
NO production by human aortic endothelial cells under glucotoxic conditions	3-HPP	1 µM	Insulin-stimulated increase in NO production was preserved, as well as phosphorylation of Akt and eNOS. The increase in ROS and RNS was prevented.	[48]
Endothelial function in human EA.hy926 endothelial cells	3,4-DHPA, 2,3-DHB and 3-HPP	10–12 µM	3,4-DHPA and a mixture of the metabolites increased the NO generation and phosphorylation of eNOS, Akt, and AMPK. Under oxidative stress, cell viability was improved by the metabolites and reduced eNOS phosphorylation was reversed. ROS generation and phosphorylation of ERK and JNK were reversed.	[49]
**Antiglycative activity**				
AGEs formation in BSA/glucose system and glyoxal trapping ability	PG, 3,4-DHPP, DHFA, 3-HPA, 3,4-DHPA and HVA	2–50 µmol/L	DHFA at 10 μmol/L significantly inhibited albumin glycation. At 2 µmol/L, a mix of 3-HPA, 3,4-DHPA, and HVA inhibited glycation. PG, 3,4-DHPP, and 3,4-DHPA had a glyoxal trapping ability of 60%, 90%, and 65%, respectively.	[50]
AGEs formation in BSA/glucose and BSA/MGO systems	3,4-DHPA, 3-HPA and HVA	1 mM	The order of AGEs’ inhibition ability was: rutin > quercetin > 3,4-DHPA > aminoguanidine > 3-HPA > HVA	[51]

^1^ 2-NBDG: 2-(N-(7-nitrobenz-2-oxa-1,3-diazol-4-yl)amino)-2-deoxyglucose; Akt: protein kinase B; AMPK: 5’ adenosine monophosphate-activated protein kinase; AP-1: activator protein 1; CaMK: Ca^2+^/calmodulin-dependent protein kinase; CaMKK: calcium/calmodulin-dependent protein kinase kinase; cAMP: cyclic adenosine monophosphate; Dex: dexamethasone; ERK: extracellular signal–regulated kinases; G-6-Pase: glucose-6-phosphatase; GLP-1: glucagon-like peptide-1; GLUT1: glucose transporter type 1; GLUT4: glucose transporter type 4; GSE: grape seed extract; GS: glycogen synthase; GSIS: glucose-stimulated insulin secretion; GSK3ß: glycogen synthase kinase 3 beta; GSPE: grape seed procyanidin extract; GTE: green tea extract; GTP: green tea polyphenol mixture; hIAPP: human islet amyloid polypeptide; IAPP: islet amyloid polypeptide; IC_50_: half maximal inhibitory concentration; IL: interleukin; IR: insulin receptor; IRS-1: insulin receptor substrate 1; JNK: c-Jun N-terminal kinases; MAPK: mitogen-activated protein kinase; MCP-1: monocyte chemoattractant protein 1; mtDNA: mitochondrial DNA; NAC: N-acetylcysteine; NF-κB: nuclear factor kappa-light-chain-enhancer of activated B cells; oxLDL: oxidized LDL; PA: palmitic acid; PEPCK: phosphoenolpyruvate carboxykinase; PGC-1: peroxisome proliferator-activated receptor coactivator-1; PI3K: phosphoinositide 3-kinase; PKC: protein kinase C; PPARγ: peroxisome proliferator-activated receptor-γ; PRC: PGC-1-related coactivator; PTP1B: protein-tyrosine phosphatase 1B; ROS: reactive oxygen species; SFA: saturated fatty acid; SOD: superoxide dismutase; STC: secretin tumor cell; TEM: transmission electron microscopy; TNFα: tumor necrosis factor; WTE: white tea extract. Flavan-3-ols and microbial metabolites: 2,3-DHB: 2,3-dihydroxybenzoic acid; 3-HB: 3-hydroxybenzoic acid; 3-HPA: 3-hydroxyphenylacetic acid; 3-HPP: 3-hydroxyphenyl propionic acid; 3-PP: 3-phenylpropionic acid; 3,4-DHPA: 3,4-dihydroxyphenylacetic-acid; 3,4-DHPP: 3,4-dihydroxyphenyl propionic acid; 4-HPA: 4-hydroxyphenylacetic acid; 4-MC: 4-methylcatechol; 5-PVA: 5-phenylvaleric acid; C: catechin; CG: catechin gallate; DHFA: dihydroferulic acid; EC: epicatechin; ECG: epicatechin gallate; ECG3”Me: epicatechin-3-*O*-(3-*O*-methyl) gallate; EGC: epigallocatechin; EGCG: epigallocatechin gallate; EGCG3”Me: epigallocatechin-3-*O*-(3-*O*-methyl) gallate; GC: gallocatechin; GCG: gallocatechin gallate; HA: hippuric acid; HVA: homovanillic acid; *m*-CoA: m-coumaric acid; PCA: protocatechuic acid; PhG: phloroglucinol; PG: pyrogallol; TF: theaflavin; TF-3-G: theaflavin-3-gallate; TF-3′-G: theaflavin-3′-gallate; TFDG: theaflavin-3,3′-digallate; VA: valeric acid; VL: valerolactone.

**Table 2:** In vivo studies on antidiabetic effects of flavan-3-ols and their microbial metabolites ^2^.

In Vivo Model	Treatment	Dose/Route/Period	Results	Ref.
**Glucose absorption in the gut**				
Inhibition of α-amylase activity in mice.	High vs. low DP proanthocyanidins	150 mg/kg/day. Oral. 56 days.	High DP proanthocyanidins had a stronger inhibition rate of digestive enzyme activity than the low DP group (0.20 ± 0.03 vs. 0.27 ± 0.06 U mg/prot in small intestine, 0.26 ± 0.04 vs. 0.35 ± 0.04 U mg/prot in pancreas)	[52]
**Insulin signaling pathways and glucose peripheral uptake**				
GLUT4 translocation in ICR mice. GLUT4 translocation in C57BL/6 mice.	CLP (EC, C, procyanidin)	250 mg/kg. Oral. Single dose. Diet with 0.5% (*w*/*w*). Oral. 7 days.	Enhanced GLUT4 translocation in skeletal muscle of ICR mice after a single dose following glucose load.	[53]
			Enhanced GLUT4 translocation in skeletal muscle of C57BL/6 mice after consecutive administration of CLP.	
GLUT-4 expression and JNK phosphorylation in KK-Ay mice.	GTP	150–300 mg/kg/day. Oral. 4 weeks.	Decreased glucose levels and enhanced glucose tolerance. JNK phosphorylation in adipose tissues was reduced and GLUT4 expression was increased. ROS content was reduced.	[26]
OGTT and GLUT4 translocation in skeletal muscle of ICR mice. AMPK activation in ICR mice.	CLP and high vs. low DP pro-cyanidins	10 mg/kg. Oral. Single dose.	Reduction of plasma glucose levels after OGTT. Promotion of GLUT4 translocation by high and low DP procyanidins. Activation of AMPK-signaling pathway. Increased phosphorylation of IRβ, IRS-1, and P13K in muscle. Low-DP increased phosphorylation of Akt.	[54]
			Increased insulin secretion in plasma.	
GLUT4 translocation in skeletal muscle of ICR mice. Activation of insulin and AMPK signaling pathways in ICR mice soleus muscle.	EC, Procyanidin B2, Procya-nidin C1, PA4-1 and cinnamtannin A2	10 μg/kg. Oral. Single dose.	Reduction of hyperglycemia after an OGTT. Increase of GLUT4 translocation.	[55]
			Promotion of AMPK, PI3K, and Akt phosphorylation.	
Glucose uptake in STZ-induced T1D Wistar/ST rats. Glucose uptake in KK-Ay mice.	GTE (C, EC, GC, EGC, CG, ECG, GCG, EGCG and caffeine)	2 g/L. Oral. 12 d. 2 g/L. Oral. 63 days vs. 42 days.	Significantly lower blood glucose level after OGTT. Increased GLUT4 translocation. Reduction in STZ-induced increase in plasma fructosamine and HbA1c.	[56]
			Significantly lower blood glucose level after OGTT. Increased GLUT4 translocation. Reduced fructosamine and HbA1c concentration.	
Glucose intolerance of HFD-induced obese and diabetic C57BL/6 mice.	EGCG	75 mg/kg. Oral. Single dose.	Blood glucose increased until 15 min (30 min in control), and rapidly decreased thereafter. It was significantly lower than in control group.	[56]
Insulin sensitivity in HFD-induced obese and diabetic C57BL/6 mice.	EC	20 mg/kg. Oral. 15 weeks.	Increase of insulin was prevented. Phosphorylation of IRS-1 and Akt was increased, while that of PKC, JNK, IKK, and PTP1B was downregulated.	[57]
IRes and effect on insulin signaling cascade in HFr-fed rats.	EC	20 mg/kg. Oral. 8 weeks.	Reversion of impaired activation of IR, IRS-1, Akt, and ERK1/2 induced by HFr diet. Reversion of upregulation of PKC, IKK, JNK, and PTP1B induced by HFr. Inhibition of HFr-induced increase of expression and activation of NADPH oxidase, expression of cytokines and chemokines, and activation of redox-sensitive signals.	[58]
Plasma glucose level in ICR mice during OGTT and GLUT4 translocation of soleus muscle.	5-(3,5-dihydroxy-phenyl)-γ-VL	32 mg/kg. Oral. Single dose.	Suppression of postprandial hyperglycemia at 15 and 30 min after OGTT. Increased GLUT4 translocation. Increased phosphorylation of AMPK.	[27]
**Beta cell viability and function**				
Glycemia, insulin, and HbA1c glycation on nicotinamide and STZ-induced diabetic rats.	EGCG	2 mg/kg. Oral. 15 days.	Glucose, HbA1c, and HOMA-IR decreased. Insulin increased.	[59]
Insulin synthesis and apoptosis in male Wistar cafeteria-induced obese rats.	GSPE	25 mg/kg. Oral. 21 days.	Decreased HOMA-IR and HOMA-ß.	[60]
			Decreased expression of Cpe.	
			Increase in Bax protein levels.	
IRes, insulin clearance, and serum lipids in NAFLD C57BL/6 mice.	EGCG	10, 20, 40 mg/kg/day. i.p. 4 weeks.	Reduction of fasting blood glucose and serum insulin. Decrease of GSIS was dose-dependently reversed. Metabolic clearance rate of insulin and IDE increased. Dose-dependent decrease of serum TC, TG, and LDL. Dose-dependent increase of serum HDL.	[61]
Antidiabetic effects in a *db/db* diabetic mouse model.	EGCG	10 g/kg diet, 1% (*w*/*w*). Oral. 10 weeks.	After an OGTT, fasting blood glucose levels decreased similarly to rosiglitazone. No changes in HOMA-IR or QUICKI. Increase in number and size of pancreatic islets comparable to rosiglitazone.	[62]
hIAPP amyloidogenesis in hIAPP transgenic mice	EGCG	0.4 mg/mL. Oral. 3 weeks.	Reduction of amyloid fibril intensity of hIAPP in the pancreas of non-diabetic transgenic mice.	[63]
Development of T1D and protective effect on pancreatic islets in NOD mice.	EGCG	0.05% in drinking water. Oral. 32 weeks.	Delayed onset of T1D. Increased plasma insulin levels compared to control. Decreased HbA1c. Increased concentration of cytokine IL-10 level. Increased islet viability when exposed to pro-inflammatory cytokines.	[64]
Immunity modulation and prevention of T1D in NOD mice.	EC	0.5% in drinking water. Oral. Single dose.	Prevention of T1D onset. Blood glucose levels decreased within the first 60 min of OGTT. HbA1c concentration reduced compared to control group.	[65]
			Plasma insulin levels were higher than in untreated group. Pancreatic islet mass improved. High proportion of immune cell-free islets. Enhanced plasma IL-10 and IL-12 levels.	
**Incretin effect**				
Plasma GLP-1 in ICR mice.	High vs. low DP pro-cyanidins	10 mg/kg. Oral. Single dose.	Increased GLP-1 secretion in plasma.	[54]
GLP-1 and plasma insulin levels in male ICR mice.	Cinnam-tannin A2	10 µg/kg. Oral. Single dose.	Increase of plasma insulin level. Increase of GLP-1 secretion levels in plasma 60 min after administration.	[66]
			Increased phosphorylation of IRß and IRS-1 in vitro in skeletal muscle.	
**Oxidative stress**				
Oxidative damage and serum lipid profile in STZ-induced diabetic rats.	C	20–80 mg/kg/day. i.p. 4 weeks.	Dose-dependent decrease of blood glucose levels. Dose-dependent increase of SOD, GST, and CAT activity. Dose-dependent decrease of TC, TG, LDL, and apoB. Dose-dependent increase of HDL and apo A-I.	[67]
Serum glucose levels and serum and hepatic biomarkers of oxidative stress in STZ-induced diabetic rats.	GTE	100 and 200 mg/kg. Oral. 4 weeks.	Decreased serum glucose levels, as well as serum and hepatic MDA concentration with 200 mg/kg for 4 weeks. TAC increased.	[68]

^2^ Akt: protein kinase B; AMPK: 5’ adenosine monophosphate-activated protein kinase; Apo: apoprotein; Bax: Bcl-2-associated X protein; bw: body weight; CAT: catalase; CLP: cacao liquor procyanidin; Cpe: carboxypeptidase E; d: day; DP: degree of polymerization; ERK: extracellular signal–regulated kinases; GLP-1: glucagon-like peptide-1; GLUT4: glucose transporter type 4; GSIS: glucose-stimulated insulin secretion; GSPE: grape seed procyanidin extract; GST: glutathione-S-transferase; GTE: green tea extract; GTP: green tea polyphenol mixture; HbA1c: glycated hemoglobin; HDL: high density lipoprotein-cholesterol; hIAPP: human islet amyloid polypeptide; HFD: high fat diet; HFr: high fructose; HOMA-IR: homeostasis model assessment of insulin resistance; HOMA-ß: homeostasis model assessment of beta cell function; i.p.: intraperitoneal; ICR: Institute of Cancer Research; IDE: insulin-degrading enzyme; IL: interleukin; IKK: IκB kinase; IR: insulin receptor; IRes: insulin resistance; IRS-1: insulin receptor substrate 1; JNK: c-Jun N-terminal kinases; LDL: low density lipoprotein-cholesterol; MDA: malondialdehyde; NAFLD: non-alcoholic fatty liver disease; NADPH: nicotinamide adenine dinucleotide phosphate; NOD: non-obese diabetic; OGTT: oral glucose tolerance test; PI3K: phosphoinositide 3-kinase; PKC: protein kinase C; PTP1B: protein-tyrosine phosphatase 1B; SOD: superoxide dismutase; STZ: streptozotocin; TAC: total antioxidant capacity; T1D: type 1 diabetes; TC: total cholesterol; TG: triglycerides; QUICKI: quantitative insulin sensitivity check index; w: week. Flavan-3-ols and their microbial metabolites: C: catechin; CG: catechin gallate; EC: epicatechin; ECG: epicatechin gallate; EGC: epigallocatechin; EGCG: epigallocatechin gallate; GC: gallocatechin; GCG: gallocatechin gallate; PA4-1: EC-(4β–6)-EC-(4β–8)-EC-(4β–8)-EC; VL: valerolactone.

**Table 3:** Human clinical trials on antidiabetic effect of flavan-3-ols ^3^.

Intervention	Study Design	Population	Duration	Parameter	Results	Ref.
**Single polyphenols**						
500 mg EGCG/day (one capsule/day) Control: 500 mg starch powder/day (one capsule/day)	CT, pc, d-b	*n* = 326 (women, GDM, 3rd trimester of pregnancy, Chinese, 25–34 years, ≈ 26kg/m^2^)	Until child’s birth	FBG, INS, HOMA-IR, QUICKI, HOMA-β, BW, BMI, neonatal complications at birth (LBW, hypoglycemia, RD, macrosomia, 1 and 5 min Apgar scores)	↓ FBG *^#^, ↓ INS *^#^, ↓ HOMA-IR *^#^, ↑ QUICKI *^#^, ↓ HOMA-β *^#^, ↓ Neonatal complications at birth	[81]
**Chocolate**						
FRC dark (100 g/day in 2 half-bar doses, 1008 mg TP, 36.12 g C). Control: FFWC	RCT, co	*n* = 19 (women 8, men 11, IGT + hypertension, 44.8 ± 8.0 years, 26.5 ± 1.9 kg/m^2^)	15 days (+ 7-day run-in and 7-day washout phase)	FBG, INS, 3-h-PBG, 3-h-PINS, HOMA-IR, β-cell function (CIR_120_), QUCIKI, ISI, lipids (TC, LDL, HDL, TG), SPB und DBP (clinical + 24-h ABMP), FMD, hsCRP, plasma homo-cysteine, electrolytes, uric acid, fibrinogen	↓ HOMA-IR *^#^, ↑ QUICKI *^#^, ↑ ISI *^#^, ↑ ISI_0_ *^#^, ↑ β-cell function (CIR_120_) *^#^ → affected 3-h-PBG and 3-h-PINS ↓ SBP *^#^ + DBP *^#^, ↓ 24-h ABMP *^#^, ↑ FMD *^#^, ↓ TC *^#^, ↓ LDL *^#^	[82]
27 g/day FRC (2 × 13.5 g, 850 mg flavan-3-ols (90 mg EC) and 100 mg IsoF	RCT, pd, pc	*n* = 93 (women, postmeno-pausal, T2D, standard therapy; receiving TC-lowering therapy, UK, ≈ 62 years, ≈ 32 kg/m^2^)	1 year	FBG, INS, HOMA-IR, HbA1c, QUICKI, lipids (TC, LDL, HDL, TG), 2-h ABPM, BW, 10-y total CHD risk	↓ INS *^#^, ↓ HOMA-IR *^#^, ↑ QUICKI *^#^, ↓ TC:HDL ratio *^#^, ↓ LDL *^#^, ↑ HDL:LDL ratio *^#^, ↑ CHD risk * but ↓ CHD risk ^#^	[83]
**GTE**						
1500 mg decaffeinated GTE (3 × 500 mg/day, 856 mg EGCG)	RCT, pc, d-b	*n* = 68 (women 44, men 24, obese T2D, Taiwanese, 51.3 ± 9.2 years, 29.7 ± 4.0 kg/m^2^)	16 weeks	FBG, INS, HOMA-IR, HbA1c, leptin, ghrelin, adiponectin, lipids (TG, TC, LDL, HDL), SPB, DBP, creatinine, ALT, uric acid, BW, BMI, WC	↓ HbA1c *, ↓ HOMA-IR *, ↓ INS *, ↑ ghrelin * (placebo too), ↓ WC *	[84]
340 mL green tea + GTE/day (582.8 mg catechins/day) Control: 340 mL green tea + GTE/day (96.3 mg catechins/day)	CT, pd, d-b	*n* = 43 (women 25, men 18, T2D, no INS therapy, hypoglycemic drugs (n = 35))	12 weeks (+ 4-week run-in, 4-week follow-up period)	FBG, INS, HbA1c, lipids (TG, TC, FFAs), total keton bodies, remnant-like lipoprotein C, adiponectin, enzymes, total protein, albumin, urea nitrogen, uric acid, creatinine, electrolytes, hematology analysis, SBP, DBP, BW, BMI, WC, HC, WHR, FM (%)	At wk 12: ↑ INS *^#^, ↑ adiponectin *, ↓ TC ^#^, ↓ FFAs *, ↓ total ketone Bodies *, ↓ WC *^#^, ↓ WHR *^#^ At wk 8: ↓ SBP *	[85]
GTE powder (one packet/day, 544 mg PP, 456 mg C)	RCT, co	*n* = 60 (women 11, men 49; glucose abnormalities, T2D medication (*n* = 16), 71% IRes, Japanese, ≈ 54 years, ≈ 26 kg/m^2^)	2 months/inter-vention	FBG, INS, HOMA-IR, HbA1c, lipids (TC, LDL, HDL, TG), hsCRP, SPB, DBP, BW, BMI, FM	↓ HbA1c *	[86]
GTE powder (one packet/day, 544 mg PP, 456 mg C)	RCT, pd	*n* = 66 (women 13, men 53; (borderline) T2D, Japanese, ≈ 54 years, ≈ 26 kg/m^2^)	2 months	FBG, INS, HOMA-IR, HbA1c, hsCRP, SPB, DBP, BW, BMI	↓ FBG, ↓ INS, ↓ HOMA-IR, ↓ HbA1c *, ↓ SPB * and DBP *, ↓ BW * and BMI *	[87]
900 mL green tea (9 g) Control: water	CT, co	*n* = 55 (women 24, men 32, T2D, 53.9 ± 7.7 years, 25.0 ± 2.2 kg/m^2^)	4 weeks	FBG, INS, HOMA-IR, adiponectin, lipids (TC, LDL, HDL, TG), hsCRP, IL-6, arterial stiffness	No effects	[88]
**GSE**						
600 mg GSE/day (2 × 300 mg/day)	RCT, co, pc, d-b	*n* = 32 (women 16, men 16, T2D at high CV risks, oral glucose-lowering therapy (n = 19), UK, 61.8 ± 6.36 years, 30.2 ± 5.92 kg⁄m^2^)	4 weeks/intervention (+2-week washout)	FBG, INS, HOMA-IR, HbA1c (only at baseline), fructosamine, lipids (TC, LDL, HDL, TG), liver function, hsCRP, endothelial function, oxidative stress (TAOS, GSH, GSSG), ACR	↓ TC *, ↓ hsCRP *, ↓ fructosamie *, ↑ GSH *	[89]
**Cacao**						
Cacao capsules (2,5 g/day ACTICOA^TM^ cacao powder, 207.5 mg Fla)	RCT, pc, pd, d-b	*n* = 35 (women 17, men 18, T2D + hypertension, dietetic and/or pharmacological treatment, 64.2 ± 1.5 years, ≈ 29 kg/m^2^	12 weeks	FBG, INS, HOMA-IR, HbA1c, lipids (TC, LDL, HDL, TG), SBP, DBD, creatinine, BW, BMI, WC, WHR, FM	↓ WC * ↓ WHR *	[90]
Cacao beverages (2 × 28 g Cacao powder/day, 180, 400 or 900 mg Fla/day) Control: cacao beverages (30 mg Fla/day)	Explora-tory rando-mized study, co	*n* = 19 (women 10, men 9, obese at risk for IRes, IGT (n = 6), 46 ± 2.3 years, 36.8 ± 1.0 kg/m^2^)	5 days (10 days washout)	FBG, INS, TG, hsCRP, ICAM, IL-6, total 8-isoprostane, SBP, DBP, BW, BMI, WC, FM After OGTT: AUC-BG, AUC-INS, 2h-PBG, 2h-PINS, 2h-TG, 2h-hsCRP, 2h-ICAM, 2h-IL-6, total 8-isopros-tane (1h and 1,5h), fibrinogen (1h and 1,5h), HOMA-IR, QUICKI, ISI	↓ 8-isoprostane *, ↓ hsCRP *, ↓ IL-6 * (as the dose of Fla increased)	[91]
Cacao beverage (960 mg PP, 480 mg Fla) with high-fat breakfast (766 kcal, 50 g fat)	RCT, pc, co, d-b (1 week washout phase)	*n* = 18 (women 14, men 4, T2D, no insulin therapy, 56 ± 3.2 years, 35.3 ± 2.0 kg/m^2^)	Single dose (6-h study: 0, 1, 2, 3, 4, 5, 6 h)	Fasting + post-prandial: BG, INS, HOMA-IR, lipids (TC, LDL, HDL, TG), hsCRP, SBP, DBP, SAE, LAE; fasting: BW, BMI, WC	↑ HDL^#^ (1 h and 4 h, 6 h-AUC, overall ∆:1.5 ± 0.8 mg/dL), ↑ Ins^#^ (4 h, overall ∆: 5.2 ± 3.2 mU/L), ↑ HOMA-IR^#^ (4 h, 4 h-AUC, no overall), ↓ LAE^#^ (2 h, overall ∆: −1.6 ± 0.7 mL/mm Hg)	[92]
2.5 g cacao (5 capsules: 0.5 g ACTICOA^TM^ cacao powder, 40.4 mg EC) with diabetic-suitable breakfast. Control: cellulose	RCT, pc, co, d-b	*n* = 12 (women 3, men 9; T2D + overweight/obesity + hypertension, no insulin therapy; 68.0 ± 9.0 years)	Single dose (4-h study: 0, 2, 4h) (≥2 week wash-out)	Fasting & post-prandial: BG, INS, HOMA-IR, lipids (TC, LDL, HDL, TG), SBP, DBP, fasting: BW, WC, HC, WHR, FM	No effects	[93]

* compared with the baseline values. ^#^ compared with the control group; ^3^ ABPM: ambulatory blood pressure monitoring; ACR: (urinary) albumin:creatinine ratio; ALT: alanine aminotransferase; AUC: area under the curve; BW: body weight; co: cross-over; CV: cardiovascular; BG: blood glucose; BMI: body mass index; CHD: coronary heart disease; CIR: corrected insulin response; CT: clinical trial; d: day; d-b: double-blind; DBP: diastolic blood pressure; FBG: fasting blood glucose; FFAs: free fatty acids; FFWC: flavanol-free white chocolate; Fla: flavanols; FM: fat mass; FMD: flow-mediated dilation; FRC: flavanol-rich chocolate; GDM: gestational diabetes mellitus; GSE: grape seed extract; GSH: reduced glutathione; GSSG: oxidized glutathione; GTE: green tea extract; HbA1c: glycated hemoglobin; HC: hip circumference; HDL: high density lipoprotein cholesterol; HOMA-IR: homeostasis model assessment for insulin resistance; hsCRP: high-sensitivity C-reactive protein; ICAM: intercellular adhesion molecule-1; IGT: impaired glucose tolerance; IL-6: interleukin-6; INS: insulin; IRes: insulin resistance; ISI: insulin sensitivity index; IsoF: isoflavones; LAE: large artery elasticity; LBW: low birth weight; LDL: low density lipoprotein cholesterol; m: men; mo: month, OGTT: oral glucose tolerance test; PBG: postprandial blood glucose; pc: placebo-controlled; pd: parallel group design; PINS: postprandial insulin concentration; PP: polyphenols; QUICKI: quantitative insulin sensitivity check index; RCT: randomized controlled trial; RD: respiratory distress; SAE: small artery elasticity; SBP: systolic blood pressure; TAOS: total antioxidant status; TC: total cholesterol; TG: triglycerides; TP: total phenols; T2D: type 2 diabetes; w: women; sig.: significant; WC: waist circumference; WHR: waist to hip ratio; wk: week; y: year. Flavan-3-ols and their microbial metabolites: EC: epicatechin; EGCG: epigallocatechin gallate.
